# Draft genome sequence of *Staphylococcus gallinarum* MTR_B001 strain isolated from breast muscle of a chicken in Bangladesh

**DOI:** 10.1128/MRA.00647-23

**Published:** 2023-10-17

**Authors:** Md. Saiful Islam, Fatimah Muhammad Ballah, M. Nazmul Hoque, A. M. M. Taufiquer Rahman, Md. Tanvir Rahman

**Affiliations:** 1 Department of Microbiology and Hygiene, Faculty of Veterinary Science, Bangladesh Agricultural University, Mymensingh, Bangladesh; 2 Department of Animal Sciences, University of California—Davis, Davis, California, USA; 3 Department of Gynaecology, Obstetrics and Reproductive Health, Bangabandhu Sheikh Mujibur Rahman Agricultural University, Gazipur, Bangladesh; 4 Naogaon District Hospital, Naogaon, Bangladesh; Indiana University, Bloomington, Indiana, USA

**Keywords:** chicken, *S. gallinarum*, WGS, antibiotic resistance genes, virulence factor genes, Bangladesh

## Abstract

We announce the genome sequence of the *Staphylococcus gallinarum* MTR_B001 strain isolated from the breast muscle of a chicken in 2022 in Bangladesh. This assembled genome had an estimated length of 2,889,393 bp (with 50× genome coverage), 15 contigs, 36 predicted antibiotic resistance genes, and 27 predicted virulence factor genes.

## ANNOUNCEMENT

*Staphylococcus gallinarum* is a frequently encountered microorganism within the surroundings, predominantly noted for its prevalence among poultry species (1). Furthermore, it has been documented that *S. gallinarum* and *S. chromogens* are implicated as the underlying cause of systemic infections in broilers (2). Although *S. gallinarum* is generally not deemed harmful to humans, instances have arisen where it was detected in wounds of hospitalized patients, in the bloodstream of an individual with chronic hepatitis B infection, and even in situations of ocular infection, particularly the condition known as endophthalmitis (3).

Between January 2022 and June 2022, chicken breast muscle samples were collected from poultry meat markets in the Mymensingh district of Bangladesh (24.7539°N, 90.4073°E) and transported to the laboratory (24.7196°N, 90.4267°E). Twenty-five grams of each sample were homogenized in 225 mL of buffered peptone broth (BPB) (HiMedia, India) and overnight incubated at 37°C. After the incubation period, the samples were streaked onto Mannitol Salt agar (HiMedia, India) plates, and the resulting colonies were subjected to staining and biochemical tests to isolate *Staphylococcus gallinarum* (4). To identify *S. gallinarum*, a matrix-assisted laser desorption ionization time-of-flight mass spectrometry was utilized (5). Subsequently, the *S. gallinarum* MTR_B001 isolate was grown aerobically on a 5% bovine blood agar plate, incubated for 24 h at 37°C, and a grown single colony was incubated in BPB. The overnight incubated broth culture was used to extract the genomic DNA in a Qiagen DNA Mini Kit (QIAGEN, Hilden, Germany). Afterward, a sequencing library was generated using the Nextera DNA Flex Library Prep Kit (Illumina, San Diego, CA, USA), and it was sequenced on the Illumina NextSeq 2000 platform using paired-end reads (2  ×  150). The raw paired-end reads (*n* = 5,704,959) of the genome were trimmed using Trimmomatic.v0.39 (6), and their quality was assessed using FastQC.v0.11.7 (7). The genome was then assembled by Unicycler.v0.4.9 (8). Following that, the genome underwent annotation utilizing PGAP.v3.0 (9). The SpeciesFinder.v2.0 (10) was used to identify the bacterial species. CARD.v3.2.4 with RGI main (11) and PATRIC.v3.2.76 (12) were employed to identify antibiotic resistance genes (ARGs), while virulence factor genes (VFGs) were determined using the Virulence Factor Database with VFanalyzer (13). Drug target genes (DTGs) were identified using DrugBank.v4.0 (14) and Therapeutic Target Database (15), transporter genes (TGs) were examined using the Transporter Classification Database (16), and metabolic functional features were assessed using RAST.v2.0 (17). Default parameters were applied for all the databases and software unless explicitly specified.

Our assembled *S. gallinarum* MTR_B001 genome consisted of 15 contigs, with two L50 contigs and an N50 value of 1,169,661 bp. The total length of the genome was 2,889,393 bp, with a genome coverage of 50.0×. The average guanine + cytosine content in the genome was 33.06%. This genome had 2,755 genes, 2,689 protein-coding sequences, 66 RNA genes, and 6 pseudogenes. The genome of the *S. gallinarum* MTR_B001 strain contained 36 predicted ARGs linked to different antibiotic classes. This genome also harbored 27 predicted VFGs associated with diverse virulence classes. Additionally, our annotated genome comprised 17 predicted DTGs and 17 predicted TGs. Furthermore, our assembled genome encompassed 283 subsystems with a coverage of 33% and 1,282 genes.

The institutional ethical committee of Bangladesh Agricultural University, Mymensingh, provided approval [AWEEC/BAU/2022 (20)] for the methodologies and corresponding protocols utilized in this study.

## Data Availability

The WGS shotgun analysis of *S. gallinarum* MTR_B001 was submitted to GenBank and assigned the accession number JAPCHT000000000. The associated information, such as the raw reads, was deposited with BioProject accession number PRJNA889186, BioSample accession number SAMN31232916, and SRA accession number SRR25296203. In this paper, the scientific version mentioned is JAPCHT000000000.1.
